# Mono-PEGylation of Alpha-MMC and MAP30 from *Momordica charantia* L.: Production, Identification and Anti-Tumor Activity

**DOI:** 10.3390/molecules21111457

**Published:** 2016-10-31

**Authors:** Yun Sun, Fenghui Sun, Jianlong Li, Minlu Wu, Xiang Fan, Yanfa Meng, Yao Meng

**Affiliations:** 1 School of Medical Laboratory Science, Chengdu Medical College, Chengdu 610500, Sichuan, China; sy83016603@163.com (Y.S.); sunfenghui_83@hotmail.com (F.S.); jlli999@foxmail.com (J.L.); wml8198@sina.com (M.W.); 2 The First Affiliated Hospital of Chengdu Medical College, Chengdu 610000, Sichuan, China; 3 Key Laboratory of Bio-Resources and Eco-Environment Ministry of Education/Animal Disease Prevention and Food Safety Key Laboratory of Sichuan Province, College of Life Science, Sichuan University, Chengdu 610064, Sichuan, China; fxscu1990@163.com (X.F.); yfmeng0902@scu.edu.cn (Y.M.)

**Keywords:** ribosome-inactivating protein, alpha-momorcharin (α-MMC), momordica anti-HIV protein (MAP30), protein PEGylation, anti-tumor

## Abstract

PEGylation is a well-established and effective strategy to decrease immunogenicity, which can increase the stability and in vivo half-life time. However, the generation of multi-site modified products is inevitable due to the lysine chemistry, which will bring difficulties in subsequent research, such as purification and quantification. Site-specific modification by mPEG-succinimidyl carbonate (mPEG-SC) is a widely used method for *N*-terminal conjugation. In this study, we used it for site-directed modification on two ribosome-inactivating proteins (RIPs), alpha-momorcharin (α-MMC) and momordica anti-HIV protein (MAP30), from *Momordica charantia* L. According to the optimization of previous modification conditions, we compared Macro-Cap SP with SP-Sepharose FF chromatography for separating the final mPEGylated RIPs. Two kinds of methods both can obtain homogenous mPEGylated RIPs which were identified by sodium dodecylsulphate polyacrylamide gel electrophoresis (SDS-PAGE), isoelectric focusing electrophoresis (IEF), and matrix-assisted laser desorption ionization-time of flight/time of flight (MALDI-TOF/TOF) analysis. We also used iodine staining method to detect the amount of unmodified PEG. Furthermore, the inhibition activity of both mPEGylated and non-PEGylated RIPs against human lung adenocarcinoma epithelial A549 cells was detected. All of the results suggested that the mPEGylated α-MMC/MAP30 might be potentially developed as new anti-tumor drugs.

## 1. Introduction

Alpha-momorcharin (α-MMC) and momordica anti-HIV protein of 30 kDa (MAP30) are both type I ribosome-inactivating proteins (RIPs) which are the members of the single chain RIP (SCRIP) family [1,2,3]. Both of these two RIPs can act irreversibly on ribosomes by removing the adenine residue from eukaryotic ribosomal 28S RNA [4]. Recently, various medicinal activities of α-MMC and MAP30 were studied, which included anti-tumor activities in vitro and in vivo. Both of them also owned broad-spectrum anti-virus activities against HSV-1, HBV, and HIV [5,6,7]. According to these findings, researchers performed plenty of studies in order to adapt them for clinical use. However, as exogenous proteins, α-MMC and MAP30 may cause serious allergic reactions, strong immunogenicity, toxic side-effects, and have short plasma half-life periods which limit their clinical applications [8,9]. To overcome these problems, our laboratory utilized different types of polyethylene glycols, especially the m(PEG)_2_-NHS, to modify α-MMC and MAP30 [9]. Although the study confirmed that m(PEG)_2_-NHS-RIPs could maintain a certain biological activity and effectively reduce the immunogenicity and side-effects, the prominent problem of this modifier was that the selectivity was not high enough and would bring out mono- or poly-PEG-RIPs compounds. Accordingly, in this manuscript, we shifted our focus on a new chemical modifier, methoxy PEG succinimidyl succinamide (mPEG-SC), which can specifically link to the *N*-terminus of proteins. Furthermore, a purification strategy for separating RIPs and their mPEG-SC-RIPs counterparts was also described here. The comparison of the final purification method by Macro-Cap SP with SP Sepharose FF can provide a good guarantee for the subsequent research. After identification of the mPEGylated RIPs, further studies about their anti-tumor activities were determined. To our knowledge, this was the first study concerning the *N*-terminal PEGlyation of both α-MMC and MAP30. The obtained mPEGylated samples through our purification strategy can be used for further research on their structure or biological function.

## 2. Results and Discussion

### 2.1. Preparation and Purification of mPEG-SC-RIPs

The solution-phase synthesis method was used in this part and the separation efficiency of Macro-Cap SP and the SP-Sepharose FF column were also compared here. In general, the PEGylation procedure for α-MMC is essentially the same as that for MAP30, except when explicitly stated. 

**Figure 1:**
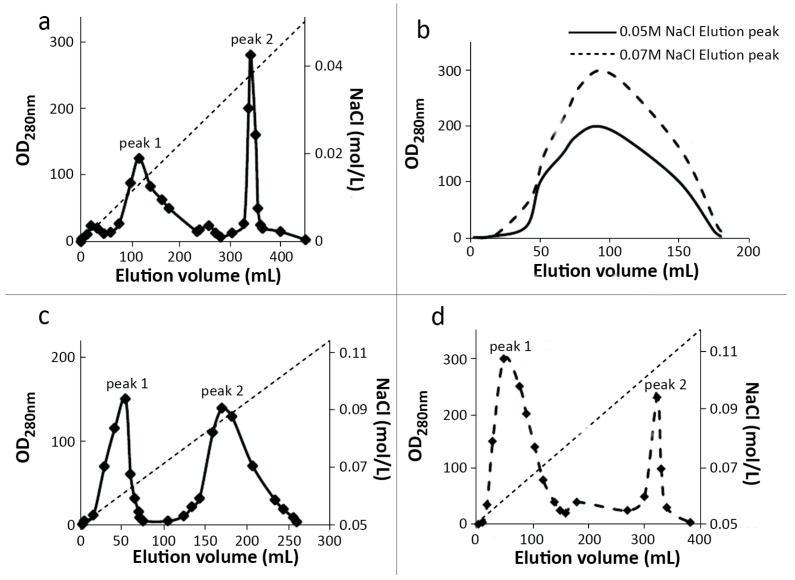
Elution profile of mono-PEGylated RIPs on Macro-Cap SP and SP-Sepharose FF column. (**a**) Chromatography of α-MMC and mono-PEGylated α-MMC fractions on Macro-Cap SP column. Peak 1 was 10 kDa-mono-PEGylated α-MMC and peak 2 was unmodified α-MMC; (**b**) chromatography of α-MMC and mono-PEGylated α-MMC fractions on SP-Sepharose FF column. 0.05 M NaCl elution peak was 10 kDa-mono-PEGylated α-MMC and 0.07 M NaCl elution peak was unmodified α-MMC; and (**c**,**d**) were the chromatography profiles of MAP30 and mono-PEGylated MAP30 fractions on the Macro-Cap SP and the SP-Sepharose FF column. Peak 1 were 10 kDa mono-PEGylated MAP30 and peak 2 were unmodified MAP30. The unconjugated mPEG-SC was washed away in the first step when adding samples to the Macro-Cap SP column or the SP-Sepharose FF column.

Under the modified conditions which was optimized in our previous research, a high PEGylation rate can be obtained and the results were summarized in Table 1. Both of these two columns can achieve the purpose in separating mPEG-SC-RIPs. When 80 mg of α-MMC or MAP30 was used as substrates, 25.34 mg of mPEGylated α-MMC and 43.1 mg of mPEGylated MAP30 can be obtained. The yields of were 31.7 % and 53.88 % in Macro-Cap SP chromatography, respectively, while in SP-Sepharose FF chromatography, 34.3 mg of mPEGylated α-MMC and 45.1 mg of mPEGylated MAP30 can be obtained. The yields of them were 42.87 % and 56.37 %, respectively. From this result, it can be implied that both Macro-Cap SP and SP-Sepharose FF chromatography methods can achieve mPEG-SC-α-MMC and mPEG-SC-MAP30. Moreover, we found that if we reduced the inner diameter of the SP-Sepharose FF column and heighten it, the separation efficiency will be further improved. The purification strategy for separating RIPs and mPEGylated RIPs described here was mainly focused on the final step in the whole process (Figure 1). Using this purification system combined with previous steps [10,11,12,13], we can obtain sufficient samples for other studies.

### 2.2. Identification of Mono-PEGylated RIPs

**Figure 2:**
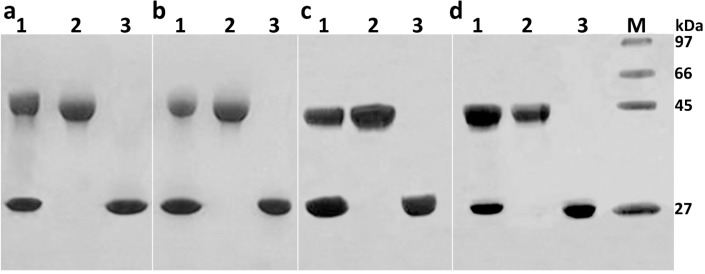
Analysis of reactive products 10 kDa mono-PEGylated α-MMC/MAP30 on SDS-PAGE. (**a**) Macro-Cap SP chromatography; lane 1: the mixture of α-MMC and mono-PEGylated α-MMC; lane 2: 10 kDa-mono-PEGylated α-MMC from peak 1 in Macro-Cap SP chromatography; lane 3: peak 2, non-reactive α-MMC in Macro-Cap SP chromatography; (**b**) SP-Sepharose FF chromatography; lane 1: the mixture of α-MMC and mono-PEGylated α-MMC; lane 2: 10 kDa mono-PEGylated α-MMC from 0.05 M NaCl elution peak in SP-Sepharose FF chromatography; lane 3: 0.07 M NaCl elution peak, non-reactive α-MMC in SP-Sepharose FF chromatography; (**c**) Macro-Cap SP chromatography; lane 1: the mixture of MAP30 and mono-PEGylated MAP30; lane 2: 10 kDa-mono-PEGylated MAP30 from peak 1 in Macro-Cap SP chromatography; lane 3: peak 2, non-reactive MAP30 in Macro-Cap SP chromatography; (**d**) SP-Sepharose FF chromatography; lane 1: the mixture of MAP30 and mono-PEGylated MAP30; lane 2: 10 kDa mono-PEGylated MAP30 from peak 1 in SP-Sepharose FF chromatography; lane 3: peak 2, non-reactive MAP30 in SP-Sepharose FF chromatography. (M). LMW (Low molecular weight) calibration kit (phophorylase, 97 kDa; albumin, 66 kDa; ovalbumin, 45 kDa; carbonic anhydrase, 27 kDa).

Applying the above purification strategy, amounts of unmodified PEGylated α-MMC/MAP30 can be removed. The collected mono-PEGylated RIPs were then analyzed by sodium dodecylsulphate polyacrylamide gel electrophoresis (SDS-PAGE), isoelectric focusing electrophoresis (IEF), and matrix-assisted laser desorption ionization-time of flight/time of flight (MALDI-TOF/TOF) analysis. From the results of SDS-PAGE (Figure 2), it was concluded that two major bands were observed in Lane 1 and those were the mixture of PEGylated RIPs and unmodified RIPs. Lane 3 represented the unmodified RIPs and its molecular weight was about 30 kDa, which was calibrated with the molecular weight marker. On the contrary, lane 2 was the PEGylated RIPs whose molecular weight was about 40 kDa.

**Figure 3:**
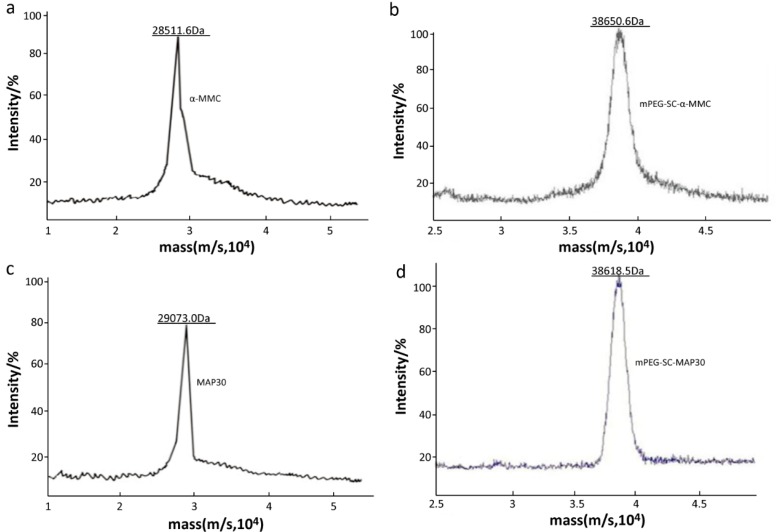
MALDI-TOF/TOF analysis of RIPs and 10 kDa mPEG-SC-RIPs. (**a**,**c**): MALDI-TOF/TOF profile of α-MMC and MAP30. The average molecular weights were 28,551.6 Da and 29,073.0 Da; (**b**,**d**): MALDI-TOF/TOF profile of 10 kDa mPEG-SC-α-MMC/MAP30. The average molecular weights were 38,650.6 Da and 38,618.5 Da.

In order to further analyze the change of molecular weight after modification, MALDI-TOF/TOF was used in this part. The accurate molecular weight was shown in Figure 3 which displayed that the molecular weights of unmodified α-MMC and MAP30 were 28,551.6 Da and 29,073.0 Da, respectively. Compared with unmodified α-MMC and MAP30, the mPEGylated counterparts were 38,650.6 Da and 38,618.5 Da, respectively. It can be indicated that the 10 kDa mPEG-SC was successfully conjugated with our purified RIPs to a certain extent.

**Figure 4:**
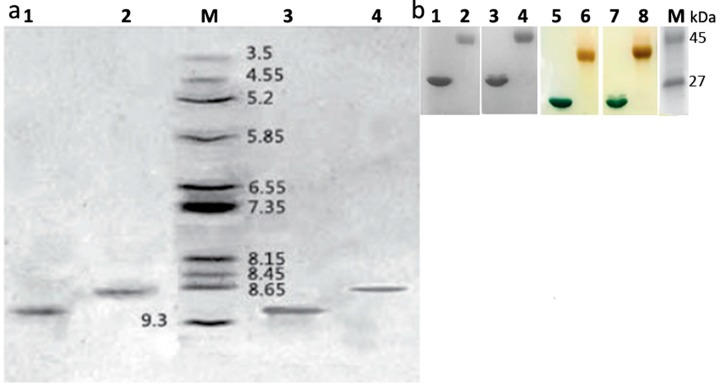
Electrophoresis analysis of RIPs and mPEG-SC-RIPs by IEF and gradient PAGE. (**a**) IEF; lane 1: MAP30; lane 2: mono-PEGylated MAP30; lane M: broad pI calibration kit with amyloglucosidase, 3.5; soybean trypsin inhibitor, 4.55; β-lactoglobulin A, 5.2; carbonic anhydrase B bovine, 5.85; carbonic anhydrase B human, 6.55; myoglobin horse, 7.35, lentil lectin-acidic band, 8.15; lentil lectin-middle band, 8.45; lentil lectin-basic band, 8.65; and trypsinogen, 9.3; lane 3: α-MMC; lane 4: mono-PEGylated α-MMC; (**b**) Gradient PAGE; lanes 1, 2, 3, and 4 represented α-MMC, mPEGylated α-MMC, MAP30, and mPEGylated MAP30 stained by Coomassie brilliant blue; lanes 5, 6, 7, and 8 represented the same while stained by iodine potassium. Lane M represented the LMW calibration kit (ovalbumin, 45 kDa; carbonic anhydrase, 27 kDa).

As for pI (isoelectric point) detection, the standard curve can be obtained according to the relationship between the isoelectric point of several standard proteins and the mobility (Figure 4a). The calculated pI of α-MMC was 9.02, mPEGylated α-MMC was 8.66, MAP30 was 9.12, and mPEGylated MAP30 was 8.67. The theoretical pI calculated with 9.02 of α-MMC and 8.86 of MAP30 was referenced with the ExPASy Proteomics Server (http://web.expasy.org/compute_pi/) and NCBI protein database (https://www.ncbi.nlm.nih.gov/protein/P16094.2; https://www.ncbi.nlm.nih.gov/protein/AAB35194.2). Herein, we can confirm that the pI of modified RIPs was lower than the unmodified ones, and this was consistent with 20 kDa m(PEG)_2_-Lys-NHS in our previous report [9]. According to these characteristics about pI, we can imply that there is a declining trend, especially when the structures of PEG was more complicated. The reason for this may be due to different lysine residues on the surface of RIPs, which will lead to a difference in the contribution to pI. In order to further identify the modification effect, gradient gel electrophoresis was performed and stained by Coomassie brilliant blue (detecting protein which contained modified RIPs and unmodified RIPs) and iodine (detecting PEG which contained modified RIPs and unconjugated PEG). From the result of Figure 4b, it displayed that the product of chemical synthesis by chromatography methods contained no residual PEG.

### 2.3. Inhibitory effect of RIPs and mPEGylated RIPs on A549 Cells

**Figure 5:**
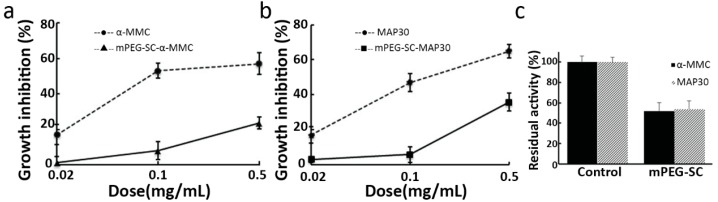
Inhibitory effects of α-MMC/MAP30 and mPEG-SC-α-MMC/MAP30 on human lung adenocarcinoma epithelial A549 cells. (**a**) A549 cells were treated with increasing concentrations of α-MMC and mPEG-SC-α-MMC for 72 h; (**b**) A549 cells were treated with increasing concentrations of MAP30 and mPEG-SC-MAP30 for 72 h; (**c**) Residual activity in comparison with α-MMC/MAP30 and mPEG-SC-α-MMC/MAP30 on A549 cells. Note: Each data point represented the average of three independent experiments performed in quadruplicate. Error bars showed standard deviations (*n* = 3). One-hundred percent of residual activity was defined as the control group to which RIPs or m-PEG-SC-RIPs were not added. *p* < 0.05 compared with control group.

In order to detect the inhibitory effect of both RIPs and mPEG-SC-RIPs, A549 cells were seeded on 96-well plates and added with increasing concentrations of purified RIPs and mPEG-SC-RIPs for 72 h (Figure 5a,b). Statistical analysis revealed a growth inhibition of A549 cells after 72 h incubation with the concentrations of 0.02, 0.1 and 0.5 mg/mL. The results exhibited that mPEGylated α-MMC and MAP30 allowed an approximately 17% activity decrease at 0.02 mg/mL tested concentration compared with non-PEGylated α-MMC and MAP30. When adding the highest concentration at 0.5 mg/mL, the activity decreased up to 40%. This demonstrated a dose-dependent inhibition behavior and can be confirmed that the anti-tumor activity against A549 cells would be decreased to a certain extent when modified with 10 kDa mPEG-SC, and the reason may due to the steric hindrance which affected the active domain. The similar results were observed when modified with 20 kDa m(PEG)_2_—Lys-NHS against human epidermal carcinoma A431 cells and immortalized human keratinocyte HaCaT cells [14], as well as JAR choriocarcinoma cells [9].

## 3. Materials and Methods

### 3.1. Materials and Reagents

*Momordica charantia* L. seeds were provided by the Institute of Agricultural Science and Technique of Sichuan Province, China. Matrices for SDS-PAGE and IEF were products of Bio-Rad Laboratories (Hercules, CA, USA). The chromatography medium for MacroCap SP was purchased from GE Healthcare Bio-Sciences AB (Uppsala, Sweden). 2-(*N*-Morpholino)ethanesulfonic acid (MES) and agarose were the products of Sigma-Aldrich (St Louis, MO, USA). mPEG-SC (10 kDa) was obtained from Shearwater Polymers (Huntsville, AL, USA). Human lung adenocarcinoma epithelial A549 cells were provided by the West China Center of Medical Sciences of Sichuan University (Chengdu, China). All other chemical reagents were standard commercial products of analytical grade.

### 3.2. Chemical Synthetic Procedure of Mono-mPEG-RIPs

In a typical preparation under optimized conditions, 10 mL (4 mg protein per milliliter) of α-MMC or MAP30 in 50 mM phosphate buffer, pH 4.0 containing 18 mM sodium cyanoborohydride was quickly mixed with 200 mg of mPEG-SC powder at a protein:PEG = 1:5 mass ratio and was slowly vibrated at 4–6 °C overnight. The modification rate was investigated by SDS-PAGE with Quality One software (Bio-Rad Laboratories) by the following formula:

$\mathrm{PEGylation}\;\mathrm{rate}\;\left(\text{\%}\right)=\left(1-\frac{the\;mass\;of\;residual\;RIPs\;in\;the\;reaction\;system}{the\;initial\;mass\;of\;RIPs\;in\;the\;reaction\;system}\right)\times 100$

### 3.3. Isolation and Purification of Mono-mPEGylated

#### 3.3.1. MacroCap SP

MacroCap^™^ SP is a cation exchanger designed to purify polyethylene glycol-modified (PEGylated) proteins and other large biomolecules, at high sample load. Mono-PEGylated proteins can be separated to high purity from oligo-PEGylated and non-PEGylated proteins. Herein, a Biomax membrane with 5 kDa was used to ultrafiltrate the modified products. The exchange buffer system was pH 6.0, 10 mM phosphate buffer and the final volume was about 10 mL. Then the samples were applied onto a MacroCap SP column (1.5 cm × 20 cm) which was pre-equilibrated with pH 6.0, 10 mM phosphate buffer at a flow rate of 1.8 mL/min. After the sample was fully inside the column, the same buffer was used to elute and remove the non-reactive PEG or other byproducts until the OD_280nm_ was lowered to baseline. The bounded unmodified α-MMC and its mPEGylated counterpart were eluted with a linear gradient from 0–0.08 M NaCl, pH 6.0, 0.01 M phosphate buffer (350 mL:350 mL). The bounded unmodified MAP30 and its mPEGylated counterpart were eluted with a linear gradient from 0.05–0.15 M NaCl, pH 6.0, and 0.01 M phosphate buffer (350 mL:350 mL). The protein elution peaks were collected by partial collectors for 4 mL/tube. According to the identified result by SDS-PAGE, the elution peak with mPEGylated-SC-α-MMC/MAP30 can be merged and the final purified sample was 10 mg/mL using Biomax 5 kDa membrane for concentration. After filtration with a 0.22 μm membrane, the aliquots with 1 mL/tube were stored at 4 °C for subsequent research.

#### 3.3.2. SP-Sepharose FF

In order to find the optimum method for purifying the modified samples, we used another ion-exchange chromatography medium. The sample treatment and elution process were basically the same as Macro-Cap SP. The column (1.5 cm × 25 cm) was pre-equilibrated with pH 6.0, 10 mM phosphate buffer at a flow rate of 0.8 mL/min. The bounded unmodified α-MMC and its mPEGylated counterpart were eluted and collected by 0.01 M phosphate buffer in pH 6.0, which contained 0.05 M and 0.07 M NaCl, respectively, while the bounded unmodified MAP30 and its mPEGylated counterpart were eluted with a linear gradient from 0.05–0.15 M NaCl, pH 6.0, 0.01 M phosphate buffer (450 mL:450 mL). The storage processes were the same as in the Macro-Cap SP.

### 3.4. Identification of mPEGylated RIPs

#### 3.4.1. Determination of Protein Content

Protein content was determined according to the method of Lowry [15] using bovine serum albumin (BSA) as a standard. The DC Protein Assay Reagents Package was purchased from Bio-Rad Laboratories (Shanghai, China).

#### 3.4.2. SDS-PAGE in the Absence of α-Mercaptoethanol

SDS-PAGE was carried out by the procedure of Laemmli [16], which used 12.0% resolving gel and a 4.0% stacking gel on a Mini Protean II apparatus (Bio-Rad Laboratories). Coomassie brilliant blue R250 was used for staining and 10.0% acetic acid was used for destaining. An LMW calibration kit was utilized as standard for molecular weight determination.

#### 3.4.3. Iodine Staining for Detecting Residual PEG

Samples were performed on 7.5%–15.0% gradient gel by the method of Niepmann [17]. The gel was taken to 5.0% BaCl_2_, the same as the stationary liquid for 15 min and washed by ddH_2_O. The potassium iodide solution was used to stain the gel until the band was clear. 

#### 3.4.4. Determination of Isoelectric Points of Samples on IEF-PAGE

The detection was performed on Bio-Rad Model II Mini IEF Cell in 5.0% polyacrylamide gel and with 2.0% ampholyte. The voltage was increased to 100 V for 15 min, to 200 V for 15 min, and to 300 V for 60 min. The gel was stained by Coomassie brilliant blue R250 and the pI was confirmed by the distance against Amersham calibration kits, pH 3.0–10.0, as standard [18,19].

#### 3.4.5. The Precise Molecular Weight of Mono-mPEGylated RIPs by MALDI-TOF/TOF

The accurate molecular weight was analyzed by MALDI-TOF/TOF (Autoflex III, Bruker Corporation) (Billerica, MA, USA) [20]. The sample, 0.1 mL with 4.0 mg/mL, was mixed with 0.1 mL of 0.2% trifluoroacetic acid (TFA). Then 0.5 μL of the above solution was mixed with 0.5 μL of the matrix solution which contained 2,6-dihydroxyacetophenone (DHAP), ethanol, and ammonium citrate. The detected sample was dried by vacuum and performed by using the positive ion detection mode. The accelerating voltage was 19.2 kV and the ion source voltage was 20 kV. Data for 2 ns pulses of 340 nm nitrogen lasers were averaged for each spectrum.

### 3.5. Inhibitory Effect of Mono-mPEGylated RIPs on A549 Cells

Human lung adenocarcinoma epithelial A549 cells were maintained in Dulbecco’s modified Eagle’s medium (DMEM) (Gibco BRL, Grand Island, NE, USA) supplemented with 10% heat-inactivated fetal bovine serum (FBS) (Gibco BRL), 100 μg/mL penicillin, and 100 U/mL streptomycin in a 5% CO_2_ incubator at 37 °C (Thermo Forma 3110, Waltham, MA, USA). 3-(4,5-dimethylthiozol-2-yl)-3,4-dipheryl tetrazolium bromide (MTT) (Sigma) was used to test cell viability and proliferation. Cell concentration was adjusted to 2.0 × 10^4^ cells/mL and then plated into 96-well plate at 100 μL/well. After 12 h of initial cell attachment, mPEGylated RIPs and their counterparts were added at final concentrations of 0.02, 0.1, and 0.5 mg/mL following incubation for 72 h (four replicas per concentration). Contrastingly, cells without adding RIPs or mPEGylated ones were used as control. Finally, 20 μL (5 mg/mL) of MTT was added to each well and the plates were incubated at 37 °C for 4 h. As for each well, 100 μL of dimethyl sulfoxide (DMSO) was added and shaking for 10 min. The optical density (OD) was measured at a wavelength of 495 nm using an enzyme-linked immunoadsorbent assay (ELISA) platereader (Model 680, Bio-Rad). A comparison of the effect of RIPs and mPEGylated RIPs to the cell viability and proliferation was analyzed and compared by the percentage of inhibition which was calculated by the formula:

$\mathrm{Inhibition}\;\mathrm{rate}\text{\%}=\frac{OD_{495control}-OD_{495sample}}{OD_{495control}}\times 100\%$

## 4. Conclusions

Recently, significant numbers of reported ribosome-inactivating proteins (RIPs) possessed ideal anti-tumor or anti-virus activities. However, strong immunogenicity or a short half-life period often limit their clinical application. Polyethylene glycol (PEG), as a water-soluble and nontoxic chemical modifier, can be used to mask the antigenic sites to prevent antibody-binding and reduce antigenicity [21,22]. It was permitted by Food and Drug Administration (FDA) for conjugating with exogenous proteins since the 1990s. Several PEG-modified protein products for medical use are now commercially available, including ADAGEN^®^ (pegademase bovine), PegIntron^®^ (PEG interferon alfa-2b), PEGylated Erythropoietin (PEG-EPO), PEGylated Granulocyte Colony Stimulating Factor (PEG-G-CSF), etc. [23]. In this manuscript, through a series of chromatography methods, homogenous α-MMC and MAP30 can be made to conjugate with PEG, and their mPEGylated counterparts are finally obtained. All of these works could help us obtain sufficient qualified samples in other studies, such as anti-tumor activities in vitro or in vivo, pharmacokinetics, toxicology, etc. To sum up, we provided a simple and reliable purification strategy for separating RIPs and their mPEGylated forms. Based on the samples obtained from this technique, α-MMC and MAP30 from *Momordica charantia* L. might be developed into a new kind of anti-tumor drug in the future.

## Figures and Tables

**Table 1:** Yields of 10 kDa mPEG-α-MMC and 10 kDa mPEG-MAP30.

Separation Methods	mPEGylation Rate (%)		Yields (%)	
	α-MMC	MAP30	α-MMC	MAP30
Macro-Cap SP	37.4 ± 2.0	50.1 ± 1.9	31.7 ± 2.5	53.88 ± 1.8
SP-Sepharose FF	43.7 ± 1.9	48.9 ± 2.4	42.87 ± 2.3	56.37 ± 2.0

Note: Values given in this table were the average of three repeats ± standard deviation.
